# Rhinoseptoplasty in children^☆^

**DOI:** 10.1016/j.bjorl.2016.04.019

**Published:** 2016-05-31

**Authors:** Claudia Pereira Maniglia, José Victor Maniglia

**Affiliations:** Faculdade de Medicina de São José do Rio Preto (FAMERP), São José do Rio Preto, SP, Brazil

**Keywords:** Nasal septum deviation, Child, Rhinoplasty, Septoplasty, Desvio de septo nasal, Criança, Rinoplastia, Septoplastia

## Abstract

**Introduction:**

Untreated septal and/or nasal pyramid deviation in children should be corrected as soon as possible, because they can result in esthetic or functional problems years later.

**Objective:**

To report the surgical experience in treating children with nasal septum and/or nasal pyramid deviation.

**Methods:**

Review of medical records of 202 children, 124 (61.4%) males and 78 (38.6%) females, between 4 and 16 years of age (*M* = 11 years) who underwent rhinoplasty and/or septoplasty in a Pediatric Otolaryngology Service of the Dept. of Otolaryngology and Head and Neck Surgery between January 1994 and January 2010.

**Results:**

Septoplasty performed in 157 cases (77.7%); rhinoseptoplasty in 23 cases (11.4%), and rhinoplasty in 22 cases (10.9%).

**Conclusion:**

Nasal changes should be corrected in children, in order to provide harmonious growth, and prevent severe sequelae found in mouth breathers.

## Resumo

**Introdução:**

Desvio do septo e/ou da pirâmide nasal em crianças, se não tratado, pode apresentar problemas estéticos ou funcionais após anos, devendo ser corrigido o quanto antes.

**Objetivo:**

Relatar experiência cirúrgica no tratamento de crianças com desvio de septo nasal e/ou pirâmide nasal.

**Método:**

Revisão de prontuários de 202 crianças, sendo 124 (61,4%) do gênero masculino e 78 (38,6%) do feminino, com idade entre 4 e 16 anos (M  =  11 anos); submetidas a rino e/ou septoplastia no período de Janeiro de 1994 a Janeiro de 2010, em um Serviço de Otorrinopediatria do Dep. de ORL e Cirurgia de Cabeça e Pescoço.

**Resultados:**

Septoplastia realizada em 157 casos (77,7%); rinosseptoplastia em 23 casos (11,4%) e rinoplastia em 22 casos (10,9%).

**Conclusão:**

Alterações nasais devem ser corrigidas em crianças, para proporcionar crescimento harmônico e evitar as graves sequelas encontradas no respirador bucal.

## Introduction

Although septal and/or nasal pyramid deviation occurs in all age groups, it is most commonly diagnosed in young adults. Its prevalence varies according to age groups.^1^, ^2^ Only a minority is diagnosed or treated in childhood, but untreated individuals may later manifest esthetic or functional problems.^3^

Children with nasal obstruction from any cause may develop serious sequelae and complications related to mouth breathing. The patency of the nasal passages allows proper growth and development of the nasomaxillary complex, and, both congenital and acquired deformities, should be corrected as early as possible. Conservative modifications to the nasal septum and the performance of osteotomies in children do not alter facial growth.^4^ The correction of other obstructions should be performed during the same operation.^5^

## Objective

To report a surgical experience in treating children with nasal septal and/or nasal pyramid deviation, and to demystify the concept that the surgical procedures recommended for septal and nasal pyramid corrections should only be indicated after 15 years of age in girls and 18 in boys.

## Methods

In accordance with the Regulations of Research on Human Subjects, Resolution 196/96 of the Ministry of Health, the present study was approved by the Research Ethics Committee of the institution under Report No. 001/2012.

A cross-sectional retrospective study was conducted to evaluate the experience of Pediatric Otorhinolaryngology Service of the Department of Otolaryngology and Head and Neck Surgery on the treatment of children with nasal septal and/or nasal pyramid deviation, regardless of the cause or etiology. In the study, we evaluated the medical records of 202 children of both genders who were referred to the Pediatric Otorhinolaryngology Service between January 1994 and January 2010.

For the selection of patient data, we followed the following inclusion criteria: pediatric age range, specific surgical procedure for septal and nasal pyramid deviation correction, either associated or not with other concomitant procedures. Exclusion criteria were the presence of corrected or unrepaired cleft lip, congenital defects of the midline, such as dermoid cyst, teratoma, encephalocele or nasal gliomas, and age older than 16 years. The following data was collected: gender, age at the time of surgery, type of nasal surgical procedure, associated procedures, outpatient revaluations and postoperative complications.

Among the 202 medical records of patients evaluated, 124 (61.4%) are males and 78 (38.6%) are females, aged between 4 and 16 years, with the average being 11 years.

Patients also underwent associated procedures, when specifically indicated, in the same surgery, including nasal turbinate surgery through intraturbinal cauterization, adeno and/or tonsillectomy, endoscopic nasal surgery and otological microsurgery with placement of ventilation tube.

## Results

Table 1 shows the relationship of surgical procedures performed during the study period. Septoplasty was the most prevalent procedure, being carried out in about 78% of cases.

**Table 1:** Distribution of surgical procedures performed.

Surgical procedures	*n* (%)
Septoplasty	157 (77.7)
Rhinoseptoplasty	23 (11.4)
Rhinoplasty	22 (10.9)
Total	202 (100.0)

Among the associated procedures, nasal turbinate surgery was the most commonly performed, in approximately 34% of cases, and otological microsurgery with placement of ventilation tube was the one of lowest prevalence (0.5%) (Table 2).

**Table 2:** Distribution of associated surgical procedures.

Associated procedures	*n* (%)
Nasal conchae surgery	68 (33.7)
Adenoidectomy	29 (14.4)
Tonsillectomy	11 (5.45)
Adenotonsillectomy	4 (2.0)
Endoscopic nasal surgery	1 (0.5)
Ontological microsurgery	1 (0.5)
Total	114 (56.4)

In accordance with the routine of the Pediatrics Otorhinology Service, the method used in all patients for the correction of septal deviation was minimally invasive, with septal mucosa hemitransfixion incision on the concave side of the deviation, 2 mm posterior to the caudal edge, and, on the caudal deviations, bilateral transfixion incision. Careful elevation of mucoperichondrium and mucoperiosteum was performed over the extent of the septum, including the highest part of the septum and the floor, exposing the nasal spine maxillary crest. Then, anterior chondrotomy of the septal deviation was performed, with subperiosteal detachment on the contralateral side. Posterior, superior and inferior chondrotomies allowed the removal of the cartilaginous septum deviation. In the presence of bone deviation, this was removed conservatively, with Jansen-Middleton forceps or a 4-mm osteotome. Repositioning of cartilage or bone parts was performed when possible.

In patients with alterations of the nasal pyramid, with or without septal deviation, intercartilaginous incisions were made with detachment of the skin tissue, to access the nasal dorsum, remove a boney hump, when present, using dorsal scissors and shaver or double-guarded osteotome. The surgery was completed with lateral and medial osteotomies. No nasal tip surgery with reduction of alar cartilages or placement of cartilage grafts was performed.

The incisions were sutured with Vicryl 4-0, with placement of “splint” mold, which was removed on the seventh day after surgery, but there was no nasal packing. Currently, “splint” molds are avoided, and mattress suture are performed.

Patients were reassessed at days 7, 30, 90 and 180 post-operatively. Relapse and/or persistence of the deviated septum was the most frequent complication (14%); there were no recorded cases of bleeding in the early or late postoperative period (Table 3).

**Table 3:** Distribution of identified complications.

Complications	*n* (%)
Septal deviation recurrence/persistence	28 (14)
Nasal pyramid deviation	8 (4)
Synechiae	4 (2)
Septal perforation	2 (1)
Infection	1 (0.5)
Bleeding	0 (0)
Hematoma	0 (0)
Abscess	0 (0)
Palatal/dental anesthesia	0 (0)
Total	21 (21.3)

Of the cases that had complications, nine were reoperated (4.45%), with seven cases being reoperated due to deviated septum (3.5%), one due to septal perforation (0.5%), and one due to synechiae (0.5%).

## Discussion

During pregnancy and childhood, several traumas occur affecting the face, and their consequences often go undetected. Thus, the diagnosis of changes in septum and nasal bone position is not often made, and the child usually is taken to a specialist only when late esthetic and/or functional problems appear.^1^, ^2^, ^3^

Congenital nasal abnormalities must undergo surgery to improve the spatial relationship between the bone and the soft tissue, to make the nose more functional, and to facilitate the normal growth stimulus. Acquired changes should also be corrected in order to avoid accommodation of soft tissues to bone malpositioning, and vicious remodelings.^4^, ^5^, ^6^

Elective rhinosurgery (septoplasty, rhinoplasty, rhinoseptoplasty) should be performed in children to correct nasal function and esthetics, aiming to restore the anatomy and promote normal development.^7^, ^8^, ^9^, ^10^ The results of reconstructive rhinoplasty performed early in life are comparable to those obtained after the end of growth. The mobilization of the nasal bones and nasal septal reconstruction can and should be performed at the same time.^11^, ^12^, ^13^, ^14^, ^15^, ^16^

Possible surgical complications described include persistence or recurrence of deviation, deviation of the nasal pyramid, with cosmetic deformity, bleeding, infection, septal hematoma, septal abscess, synechia, septal perforation, palatal and dental anesthesia.^17^ Changes in cartilaginous structure, which are more vulnerable in the phases of growth, can lead to a loss of the good results achieved after initial surgery, due to puberty growth spurt, leading to postsurgical distortion. Thus, the possibility of inappropriate growth of the septum with septal deviation after surgery, and necessity of a secondary surgery in the future, should always be clarified.^5^

Nasal air flow is crucial for facial balance and good nasal physiological function. Facial deformity is not dependent on the obstruction cause and age at onset. However, the severity, duration and age for correction are essential for the settlement of possible impact on craniofacial growth.^18^, ^19^

## Conclusions

Our surgical experience of the correction of nasal septum and/or nasal pyramid deviation in children leads us to conclude that, based on clinical improvement and physical examination, these changes should be corrected as soon as possible to provide harmonious growth and prevent serious sequelae found in mouth breathers.

The nasal surgery that is performed early in children is a safe procedure, provided that it is carried out carefully, with appropriate conservation of cartilage, and respect for facial growth centers. The fear of interrupting facial growth can perpetuate the functional and esthetic problems.

## Conflicts of interest

The authors declare no conflicts of interest.
